# A Study of Physical Layer Security in SWIPT-Based Decode-and-Forward Relay Networks with Dynamic Power Splitting

**DOI:** 10.3390/s21175692

**Published:** 2021-08-24

**Authors:** Van-Duc Phan, Tan N. Nguyen, Anh Vu Le, Miroslav Voznak

**Affiliations:** 1 Faculty of Automobile Technology, Van Lang University, Ho Chi Minh City 700000, Vietnam; duc.pv@vlu.edu.vn; 2 Wireless Communications Research Group, Faculty of Electrical and Electronics Engineering, Ton Duc Thang University, Ho Chi Minh City 700000, Vietnam; 3 Optoelectronics Research Group, Faculty of Electrical and Electronics Engineering, Ton Duc Thang University, Ho Chi Minh City 700000, Vietnam; leanhvu@tdtu.edu.vn; 4 Faculty of Electrical Engineering and Computer Science, VSB-Technical University of Ostrava, 17. listopadu 2172/15, 708 00 Ostrava, Czech Republic; miroslav.voznak@vsb.cz

**Keywords:** decode-and-forward, outage probability, relay selection, secrecy outage probability, SWIPT

## Abstract

In this paper, we study the physical layer security for simultaneous wireless information and power transfer (SWIPT)-based half-duplex (HD) decode-and-forward relaying system. We consider a system model including one transmitter that tries to transmit information to one receiver under the help of multiple relay users and in the presence of one eavesdropper that attempts to overhear the confidential information. More specifically, to investigate the secrecy performance, we derive closed-form expressions of outage probability (OP) and secrecy outage probability for dynamic power splitting-based relaying (DPSBR) and static power splitting-based relaying (SPSBR) schemes. Moreover, the lower bound of secrecy outage probability is obtained when the source’s transmit power goes to infinity. The Monte Carlo simulations are given to corroborate the correctness of our mathematical analysis. It is observed from simulation results that the proposed DPSBR scheme outperforms the SPSBR-based schemes in terms of OP and SOP under the impact of different parameters on system performance.

## 1. Introduction

The Internet of Things (IoT) has become an importation application in 5G and beyond network [1,2,3,4,5,6]. With the evolution of IoT, there will be billions of connected IoT users (IoTUs), which can provide various applications, e.g., smart city, health, and agriculture. However, conventional IoTUs usually have limited operation time due to limited battery capacity. Thanks to the recent development of energy harvesting, IoTUs can help to solve the energy problem. Besides, energy sources from the ambient environment such as wind [7,8], vibration [9,10], and solar [11] can also contribute to solving the energy problem. Nevertheless, these energy sources are unreliable and depend on on external conditions, e.g., weather. Consequently, wireless power transfer (WPT) has emerged as a solution to provide a robust energy supply because the IoTUs can harvest energy from radio frequency (RF) signals, which does not influenced by surrounding environment [12,13,14,15].

Particularly, simultaneous wireless information and power transfer (SWIPT) technology has received significant attention from researchers due to its ability to bring both information and energy at the same time [16,17,18,19,20]. Specifically, the authors of [16,17] investigated SWIPT in IoT networks. Lu et al. [16] proposed two SWIPT-based spectrum sharing methods to enhance the spectrum and energy efficiency in 6G IoT networks. Wang et al. [17] investigated a SWIPT-based massive multiple-input multiple-output (MIMO) two-way relaying system by considering maximum ratio combining and zero-forcing. In [18,19,20,21], the authors investigated the SWIPT in two-way (TW) relaying networks. Nguyen et al. [21] proposed and investigated a new system model for SWIPT-based TW relaying systems. Specifically, they derived the closed-form of three relay schemes, termed decode-and-forward (DF), amplify-and-forward (AF), and hybrid decode-and-forward (HDAF). Garg et al. [18] considered the hordal distance (CD) decomposition-based precoder design to reduce the complexity as compared with semi-definite relaxation (SDR)-based methods, for the SWIPT-assisted AF TW relay system. Tin et al. [19] proposed a new EH-based TW half-duplex (HD) relay sensor network under the presence of a direct link between the transmitter and receiver. Specifically, they derived the closed-form expressions the exact and asymptotic of ergodic capacity and the exact analysis of symbol error rate. Zhang et al. [20] studied the neural network-based relay selection in SWIPT-enabled TW cognitive radio networks (CRNs). Concretely, they proposed two relay selection methods corresponding to fixed and variable number of relays, which outperformed the traditional relay selection and machine learning schemes.

Besides energy harvesting, communication security is crucially important for wireless systems. Recently, physical layer security (PLS) has become an effective method to improve the secrecy of wireless communications without sharing security keys [22,23,24,25]. In [22], the authors proposed a generalized partial relay selection (PRS) protocol to improve the security for CRNs under perfect or imperfect CSI. The PLS in millimeter-wave (mmWave) communications was investigated in [23]. Concretely, they proposed a Sight-based Cooperative Jamming (SCJ) method to enhance the secrecy performance of mmWave communications. Wijewardena studied the PLS for intelligent reflecting surface (IRS) two-way communications. In detail, they aimed to maximize the sum-secrecy rate of an IRS-assisted full-duplex (FD) TW communication system in the presence of an untrusted user. In [25], the authors considered a novel system model for EH-based PLS multi-hop multi-path cooperative wireless networks. Then, they proposed three relay protocols—shortest path, random path, and best path selection schemes—to enhance the PLS performance.

Recently, EH and SWIPT have become hot topics [26,27,28,29,30,31]. An et al. [26] considered a hybrid time-power splitting (HTPS) TW HD cooperative relaying in the presence of an eavesdropper. In this context, they derived the closed-form expression of the outage probability (OP) and intercept probability (IP) using maximal ratio combining (MRC) and selection combining (SC). The authors of [27] investigated the PLS of a power beacon-assisted FD EH relaying system using delay-tolerant (DTT) and delay-limited (DLT) methods. In [28], the PLS was studied in mmWave- and SWIPT-enabled UAV networks by considering considering actual 3-D antenna gain and the effect of beamforming design. In [29], the secrecy performance was investigated in a UAV-assisted NOMA system with SWIPT by using artificial jamming and NOMA information. The PLS of a downlink (DL) multiuser orthogonal frequency division multiplexing (OFDM) IoT system was exploited in [30]. Deng et al. [31] studied the secure beamforming design for a TW CR IoT network with SWIPT by maximizing the secrecy capacity for primary users by designing the beamforming matrix.

In this work, we proposed and investigate the secrecy performance of a SWIPT-assisted HD DF relaying system in the presence of a eavesdropper. The contributions can be summarized as follows.

- We consider a single-input single-output (SISO) system model in which multiple relay nodes harvest energy from a transmitter S and help S to transfer information to the destination in the presence of an eavesdropper. Moreover, partial relay selection protocol is adopted to select the best relay.
- For the SWIPT technique, both dynamic power splitting-based relaying (DPSBR) and static power splitting-based relaying (SPSBR) are considered in our work to give a full picture of the advantages of each method. Specifically, we derive the closed-form expressions in terms of OP and SOP for each scheme. Furthermore, the lower bound of SOP is obtained when the transmit power of S goes to infinity.
- Simulation results are performed to corroborate the exactness of our analysis. Through simulation results, it can be concludes that that DPSBR always obtains a better performance, i.e., OP and SOP, compared to SPSBR.

## 2. System Model

**Figure 1:**
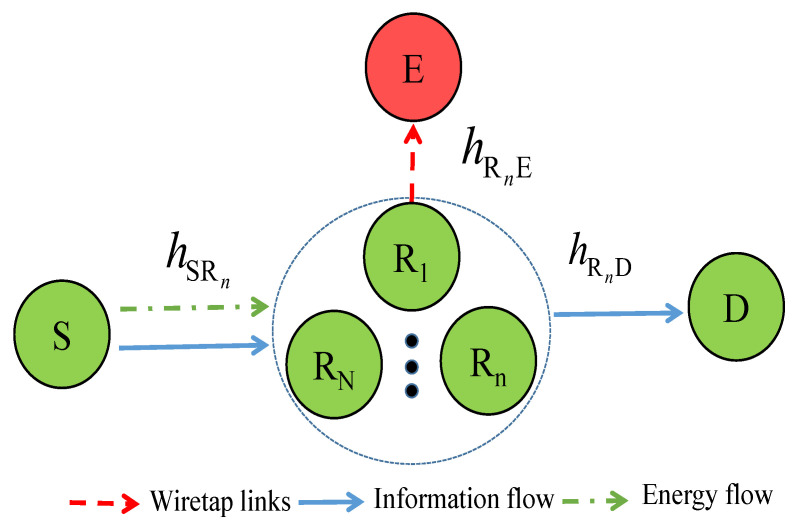
SWIPT-based cooperative relay networks in the presence of an eavesdropper.

In Figure 1, we describe the proposed system model as follows. The system includes a source S that communicates with a destination D via help of *N* half-duplex relays denoted by $R_{n}$, where n=1,⋯,N. Besides, there exists an eavesdropper that tries to overhear confidential information from relays. Moreover, the source can transmit both data and power to the relay using the SWIPT technique. As relay users are equipped with energy harvesters, they can thus harvest energy from the source’s signals and then use it to transfer information to the destination D.

Let us denote $h_{{\mathrm{SR}}_{n}}$, $h_{R_{n}D}$, and $h_{R_{n}E}$ as the channel coefficients of the $S\to R_{n}$, $R_{n}\to D$ and $R_{n}\to E$ links, respectively.

Assume that all of the channels are Rayleigh fading, thus the channel gains $\gamma_{SR_{n}}=|h_{SR_{n}}{|}^{2}$, $\gamma_{R_{n}D}=|h_{R_{n}D}{|}^{2}$, and $\gamma_{R_{n}E}={\left|h_{R_{n}E}\right|}^{2}$ are exponential random variables (RVs) whose CDF are given as ([32], Equation (1))

(1) $$\begin{array}{cc} F_{\gamma_{SR_{n}}}\left(x\right) & =1-\exp\left(-\lambda_{SR_{n}}x\right), \end{array}$$

(2) $$\begin{array}{cc} F_{\gamma_{R_{n}D}}\left(x\right) & =1-\exp\left(-\lambda_{R_{n}D}x\right), \end{array}$$

(3) $$\begin{array}{cc} F_{\gamma_{R_{n}E}}\left(x\right) & =1-\exp\left(-\lambda_{R_{n}E}x\right). \end{array}$$

To take path-loss into account, we can model the parameters as follows:

(4) $$\begin{array}{c} \lambda_{SR_{n}}={\left(d_{SR_{n}}\right)}^{\beta },\lambda_{R_{n}D}={\left(d_{R_{n}D}\right)}^{\beta },\lambda_{R_{n}E}={\left(d_{R_{n}E}\right)}^{\beta }. \end{array}$$

where $d_{SR_{n}}$, $d_{R_{n}D}$, and $d_{R_{n}E}$ are link distances of the $S\to R_{n}$, $R_{n}\to D$, and $R_{n}\to E$ links, respectively.

Then, the PDFs of $\gamma_{SR_{n}}$, $\gamma_{R_{n}D}$, and $\gamma_{R_{n}E}$ are, respectively, given as ([32] Equation (2))

(5) $$\begin{array}{cc} f_{\gamma_{SR_{n}}}\left(x\right) & =\lambda_{SR_{n}}\exp\left(-\lambda_{SR_{n}}x\right), \end{array}$$

(6) $$\begin{array}{cc} f_{\gamma_{R_{n}D}}\left(x\right) & =\lambda_{R_{n}D}\exp\left(-\lambda_{R_{n}D}x\right), \end{array}$$

(7) $$\begin{array}{cc} f_{\gamma_{R_{n}E}}\left(x\right) & =\lambda_{R_{n}E}\exp\left(-\lambda_{R_{n}E}x\right). \end{array}$$

The received signal at the relay n-th can be expressed as

(8) $$\begin{array}{c} y_{R_{n}}=\sqrt{1-\rho }h_{SR_{n}}x_{S}+n_{R_{n}}, \end{array}$$

where $x_{s}$ is the energy symbol with $E\left\{{\left|x_{S}\right|}^{2}\right\}=P_{S}$, and $E\left\{•\right\}$ denotes the expectation operation; $n_{R_{n}}$ is the zero-mean additive white Gaussian noise (AWGN) with variance $N_{0}$.

The energy harvesting in relay can be computed as (Equation (3) [33])

(9) $$\begin{array}{c} P_{R_{n}}=\frac{E_{n}}{T/2}=\eta \rho P_{S}\gamma_{SR_{n}}, \end{array}$$

where $P_{S}$ and $P_{R}$ are the transmit powers of S and $R_{n}$, respectively.

The received signal at the destination can be given as

(10) $$\begin{array}{c} y_{D}=h_{R_{n}D}x_{R_{n}}+n_{D}, \end{array}$$

where $n_{D}$ is the zero mean AWGN with variance $N_{0}$.

In this paper, we consider the DF relaying protocols. From (8), the signal to noise ratio (SNR) at the relay n-th node can be derived by

(11) $$\begin{array}{c} \gamma_{R_{n}}=\frac{\left(1-\rho \right)\gamma_{SR_{n}}P_{S}}{N_{0}}=\left(1-\rho \right)\gamma_{SR_{n}}\Phi , \end{array}$$

where $\Phi =\frac{P_{S}}{N_{0}}$.

From (9), the SNR at the destination can be obtained as

(12) $$\begin{array}{c} \gamma_{D}=\frac{P_{R_{n}}\gamma_{R_{n}D}}{N_{0}}=\eta \rho \Phi \gamma_{SR_{n}}\gamma_{R_{n}D}, \end{array}$$

Finally, the overall SNR and the capacity of system can be claimed by, respectively,

(13) $$\begin{array}{cc} \psi_{\mathrm{DF}} & =\min\left(\gamma_{R_{n}},\gamma_{D}\right), \end{array}$$

(14) $$\begin{array}{cc} C_{\mathrm{DF}} & =\frac{1}{2}\log_{2}\left(1+\psi_{\mathrm{DF}}\right). \end{array}$$

Taking into account the impact of eavesdropper E, E will overhear the information from chosen relay *n*-th, so the received signal at E can be expressed by

(15) $$\begin{array}{c} y_{E}=h_{R_{n}E}x_{R_{n}}+n_{E}, \end{array}$$

where $E\left\{{\left|x_{R_{n}}\right|}^{2}\right\}=P_{R_{n}}$ and $n_{E}$ is the zero-mean AWGN with variance $N_{0}$.

From (9) and (15), the SNR and capacity of E can be found as, respectively,

(16) $$\begin{array}{cc} \gamma_{E} & =\eta \rho \Phi \gamma_{SR_{n}}\gamma_{R_{n}E}, \end{array}$$

(17) $$\begin{array}{cc} C_{E} & =\frac{1}{2}\log_{2}\left(1+\gamma_{E}\right). \end{array}$$

**Remark** **1.** *In this work, we adopt partial relay selection (PRS) protocol. Without loss of generality, we assume that the relay is closer to source S than to destination D, and the relay selection should be performed based on the quality of the second-hop links to improve overall performance. Specifically, the best relay user is selected according to the optimal relay selection method, which is described as follows:*

(18) $$\begin{array}{c} R_{a}:\gamma_{R_{a}D}=\max_{n=1,2,...,N}\left(\gamma_{R_{n}D}\right). \end{array}$$

The cumulative density function (CDF) between selected relay $R_{a}$ to destination D, i.e., $F_{\gamma_{R_{a}D}}\left(x\right)$, is given as ([34] Equation (14))

(19) $$\begin{array}{c} F_{\gamma_{R_{a}D}}\left(x\right)=\mathrm{Pr}\left(\gamma_{R_{a}D}<x\right)=\prod_{n=1}^{N}F_{\gamma_{R_{n}D}}\left(x\right). \end{array}$$

Considering the i.i.d. random variables (RVs), i.e., $\lambda_{R_{n}D}=\lambda_{\mathrm{RD}},\forall n$, Equation (13) can be rewritten as

(20) $$\begin{array}{cc} F_{\gamma_{R_{a}D}}\left(x\right) & ={\left[1-\exp\left(-\lambda_{\mathrm{RD}}x\right)\right]}^{N}\\ & =1+\sum_{k=1}^{N}{\left(-1\right)}^{k}C_{N}^{k}\exp\left(-k\lambda_{\mathrm{RD}}x\right), \end{array}$$

where $C_{N}^{k}=\frac{N!}{k!(N-k)!}.$

Based on (20), the probability density function (PDF) of $\gamma_{R_{a}D}$, i.e., $f_{\gamma_{R_{a}D}}\left(x\right)$, can be calculated as

(21) $$\begin{array}{cc} f_{\gamma_{R_{a}D}}\left(x\right) & =\frac{\partial F_{\gamma_{R_{a}D}}\left(x\right)}{\partial x}\\ & =\sum_{k=1}^{N}{\left(-1\right)}^{k+1}C_{N}^{k}k\lambda_{\mathrm{RD}}\exp\left(-k\lambda_{\mathrm{RD}}x\right). \end{array}$$

## 3. Performance Analysis

This section provides the mathematical analysis of the outage probability (OP) and secrecy outage probability (SOP) to provide further insight into the two-hop data transmission for SWIPT-based HD DF relay networks in two cases, i.e., dynamic power splitting-based relaying and static power splitting-based relaying.

### 3.1. Case 1: Static Power Splitting-Based Relaying

#### 3.1.1. Outage Probability (OP) Analysis

In this section, the outage probability of the SWIPT-aided HD DF relaying system over Rayleigh fading channels is derived. Specifically, it can be calculated as (Equation (21) [35])

(22) $$\begin{array}{c} \mathrm{OP}=\mathrm{Pr}\left(C_{DF}<C_{th}\right)=\mathrm{Pr}\left(\psi_{DF}<\gamma_{th}\right), \end{array}$$

where $\gamma_{th}=2^{2C_{th}}-1$, and $C_{th}$ is the threshold rate at the destination to decode signals successfully.

**Theorem** **1.** *In static power splitting-based relaying, the closed-form expression of the OP can be given as*

(23) $$\begin{array}{cc} \mathrm{OP} & \approx 1+\sum_{k=1}^{N}{\left(-1\right)}^{k}C_{N}^{k}\exp\left(-k\lambda_{\mathrm{RD}}\xi -\lambda_{SR_{a}}\vartheta \right)\\ \; & +\sum_{k=1}^{N}\sum_{m=1}^{M}\frac{\pi {\left(-1\right)}^{k}C_{N}^{k}k\lambda_{\mathrm{RD}}\xi }{2M}\sqrt{1-\mu_{m}^{2}}\\ \; & \exp\left(-\frac{k\lambda_{\mathrm{RD}}\xi }{2}-\frac{\lambda_{SR_{a}}\gamma_{th}}{\eta \rho \Phi \Delta (\theta_{m})}-\frac{k\lambda_{\mathrm{RD}}\xi \theta_{m}}{2}\right), \end{array}$$

*where M determines the trade-off between complexity and accuracy for the Gaussian–Chebyshev quadrature-based approximation, where $\mu_{m}=\cos\left(\frac{\pi (2m-1)}{2M}\right)$ and $\theta_{m}=\frac{\xi }{2}\mu_{m}+\frac{\xi }{2}$.*

**Proof.**  Based on (11) and (12), OP can be expressed as

(24) $$\begin{array}{cc} \mathrm{OP} & =\mathrm{Pr}\left(\min\left(\left(1-\rho \right)\gamma_{SR_{a}}\Phi ,\eta \rho \Phi \gamma_{SR_{a}}\gamma_{R_{a}D}\right)<\gamma_{th}\right)\\ \; & =1-\mathrm{Pr}\left(\left(1-\rho \right)\gamma_{SR_{a}}\Phi \geq \gamma_{th},\eta \rho \Phi \gamma_{SR_{a}}\gamma_{R_{a}D}\geq \gamma_{th}\right)\\ \; & =1-\mathrm{Pr}\left(\gamma_{SR_{a}}\geq \frac{\gamma_{th}}{(1-\rho )\Phi },\gamma_{SR_{a}}\gamma_{R_{a}D}\geq \frac{\gamma_{th}}{\eta \rho \Phi }\right)\\ \; & =1-\int_{0}^{\xi }f_{\gamma_{R_{a}D}}\left(y\right)dy\int_{\frac{\gamma_{th}}{\eta \rho \Phi y}}^{\infty }f_{\gamma_{SR_{a}}}\left(x\right)dx\\ & -\int_{\xi }^{\infty }f_{\gamma_{R_{a}D}}\left(y\right)dy\int_{\vartheta }^{\infty }f_{\gamma_{SR_{a}}}\left(x\right)dx, \end{array}$$

where $\vartheta =\frac{\gamma_{th}}{(1-\rho )\Phi },\xi =\frac{\left(1-\rho \right)}{\eta \rho }$.

By applying (20) and (21), OP can be represented as

(25) $$\begin{array}{cc} \mathrm{OP} & =1+\sum_{k=1}^{N}{\left(-1\right)}^{k}C_{N}^{k}\exp\left(-k\lambda_{\mathrm{RD}}\xi -\lambda_{SR_{a}}\vartheta \right)\\ \; & +\sum_{k=1}^{N}{\left(-1\right)}^{k}C_{N}^{k}k\lambda_{\mathrm{RD}}\int_{0}^{\xi }\exp\left(-\frac{\lambda_{SR_{a}}\gamma_{th}}{\eta \rho \Phi y}-k\lambda_{\mathrm{RD}}y\right)dy. \end{array}$$

As it is difficult to find the closed-form expression for (25) due to the integral $\int_{m_{1}}^{m_{2}}\exp\left(\frac{v_{1}}{x}\right)$$\exp(v_{2}x)dx$, thus we will adopt the Gaussian–Chebyshev quadrature. First, we change the variable from (25) by denoting $y=\frac{\xi }{2}x+\frac{\xi }{2}$. Equation (25) can be rewritten as

(26) $$\begin{array}{cc} \mathrm{OP} & =1+\sum_{k=1}^{N}{\left(-1\right)}^{k}C_{N}^{k}\exp\left(-k\lambda_{\mathrm{RD}}\xi -\lambda_{SR_{a}}\vartheta \right)\\ \; & +\sum_{k=1}^{N}\frac{{\left(-1\right)}^{k}C_{N}^{k}k\lambda_{\mathrm{RD}}\xi }{2}\exp\left(-\frac{k\lambda_{\mathrm{RD}}\xi }{2}\right)\\ & \int_{-1}^{1}\exp\left(-\frac{\lambda_{SR_{a}}\gamma_{th}}{\eta \rho \Phi \Delta (x)}-\frac{k\lambda_{\mathrm{RD}}\xi x}{2}\right)dx, \end{array}$$

where $\Delta \left(x\right)=\frac{\xi }{2}x+\frac{\xi }{2}$.

By applying the Gaussian–Chebyshev quadrature in [36] (Equation (37), Equation (26) can be obtained as in (23), which finishes the proof. □

#### 3.1.2. Secrecy Outage Probability (SOP) Analysis

**a.**  **Exact Analysis**

The SOP can be defined as (Equation (33) [37])

(27) $$\begin{array}{c} \mathrm{SOP}=\mathrm{Pr}\left(C_{\sec}<C_{th}\right)=\mathrm{Pr}\left(\frac{1+\psi_{DF}}{1+\gamma_{E}}<\gamma_{th}\right), \end{array}$$

where $C_{\sec}=\max\left\{C_{DF}-C_{E},0\right\}$.

By substituting (13), (15), (16), and (17) into (27), we have

(28) $$\begin{array}{cc} \; & \mathrm{SOP}\\ & =\mathrm{Pr}\left(\frac{1+\min\left(\left(1-\rho \right)\gamma_{SR_{a}}\Phi ,\eta \rho \Phi \gamma_{SR_{a}}\gamma_{R_{a}D}\right)}{1+\eta \rho \Phi \gamma_{SR_{a}}\gamma_{R_{a}E}}<\gamma_{th}\right)\\ \; & =\mathrm{Pr}\left(\Phi \gamma_{SR_{a}}\min\left\{1-\rho ,\eta \rho \gamma_{R_{a}D}\right\}<\gamma_{th}-1+\chi_{1}\gamma_{SR_{a}}\right)\\ \; & =\mathrm{Pr}\left(\gamma_{R_{a}D}>\frac{1-\rho }{\eta \rho },\Phi \left(1-\rho \right)\gamma_{SR_{a}}<\gamma_{th}-1+\chi_{1}\gamma_{SR_{a}}\right)\\ \; & +\mathrm{Pr}\left(\gamma_{R_{a}D}\leq \frac{1-\rho }{\eta \rho },\Phi \left(1-\rho \right)\gamma_{SR_{a}}\gamma_{R_{a}D}<\gamma_{th}-1+\chi_{1}\gamma_{SR_{a}}\right)\\ \; & =\mathrm{Pr}\left(\gamma_{R_{a}D}>\frac{1-\rho }{\eta \rho },\gamma_{SR_{a}}\left(\Phi \left(1-\rho \right)-\chi_{1}\right)<\gamma_{th}-1\right)\\ \; & +\mathrm{Pr}\left(\gamma_{R_{a}D}\leq \frac{1-\rho }{\eta \rho },\gamma_{SR_{a}}\left(\Phi \eta \rho \gamma_{R_{a}D}-\chi_{1}\right)<\gamma_{th}-1\right). \end{array}$$

where $\chi_{1}≜\gamma_{th}\eta \rho \Phi \gamma_{R_{a}E}$.

It is noted that $\gamma_{th}=2^{2C_{th}}-1\geq 0$. Thus, (28) is reformulated as

(29) $$\begin{array}{cc} \mathrm{SOP} & =\underset{\Theta_{1}}{\underset{︸}{\mathrm{Pr}\left(\gamma_{R_{a}D}>\frac{1-\rho }{\eta \rho },\gamma_{R_{a}E}>\frac{1-\rho }{\eta \rho \gamma_{th}}\right)}}\\ & +\underset{\Theta_{2}}{\underset{\Theta }{\mathrm{Pr}\left(\gamma_{R_{a}D}>\frac{1-\rho }{\eta \rho },\gamma_{R_{a}E}<\frac{1-\rho }{\eta \rho \gamma_{th}},\gamma_{SR_{a}}<\frac{\gamma_{th}-1}{\Phi \left(1-\rho \right)-\chi_{1}}\right)}}\\ \; & +\underset{\Theta_{3}}{\underset{︸}{\mathrm{Pr}\left(\gamma_{R_{a}D}<\frac{1-\rho }{\eta \rho },\gamma_{R_{a}E}>\frac{\gamma_{R_{a}D}}{\gamma_{th}}\right)}}\\ & +\underset{\Theta_{4}}{\underset{︸}{\mathrm{Pr}\left(\gamma_{R_{a}D}>\frac{1-\rho }{\eta \rho },\gamma_{R_{a}E}<\frac{\gamma_{R_{a}D}}{\gamma_{th}},\gamma_{SR_{a}}<\frac{\gamma_{th}-1}{\Phi \eta \rho \gamma_{R_{a}D}-\chi_{1}}\right)}}. \end{array}$$

Based on (20) and (21), $\Theta_{1},\Theta_{2},\Theta_{3}$, and $\Theta_{4}$ in (29) are, respectively, calculated as

(30) $$\begin{array}{cc} \Theta_{1} & =\left\{1-\mathrm{Pr}\left(\gamma_{R_{a}D}\leq \frac{1-\rho }{\eta \rho }\right)\right\}\times \left\{1-\mathrm{Pr}\left(\gamma_{R_{a}E}\leq \frac{1-\rho }{\eta \rho \gamma_{th}}\right)\right\}\\ \; & =\sum_{k=1}^{N}{\left(-1\right)}^{k}C_{N}^{k}\exp\left[\frac{\left(\rho -1\right)}{\eta \rho }\left(k\lambda_{\mathrm{RD}}+\frac{\lambda_{{}_{R_{a}E}}}{\gamma_{th}}\right)\right], \end{array}$$

(31) $$\begin{array}{cc} \Theta_{2} & =\int_{\frac{1-\rho }{\eta \rho }}^{\infty }f_{\gamma_{R_{a}D}}\left(x\right)dx\int_{0}^{\frac{1-\rho }{\eta \rho \gamma_{th}}}f_{\gamma_{R_{a}E}}\left(y\right)dy\int_{0}^{\varphi }f_{\gamma_{SR_{a}}}\left(z\right)dz\\ \; & =\int_{\frac{1-\rho }{\eta \rho }}^{\infty }f_{\gamma_{R_{a}D}}\left(x\right)dx\int_{0}^{\frac{1-\rho }{\eta \rho \gamma_{th}}}\lambda_{R_{a}E}\left\{1-e^{\left(-\varphi \lambda_{SR_{a}}\right)}\right\}e^{\left(-\lambda_{R_{a}E}y\right)}dy\\ \; & =\sum_{k=1}^{N}{\left(-1\right)}^{k+1}C_{N}^{k}k\lambda_{\mathrm{RD}}\lambda_{R_{a}E}\\ & \times \int_{\frac{1-\rho }{\eta \rho }}^{\infty }\int_{0}^{\frac{1-\rho }{\eta \rho \gamma_{th}}}\left\{1-e^{\left(-\varphi \lambda_{SR_{a}}\right)}\right\}\times e^{\left(-k\lambda_{\mathrm{RD}}x-\lambda_{R_{a}E}y\right)}dxdy, \end{array}$$

(32) $$\begin{array}{cc} \Theta_{3} & =\int_{0}^{\frac{1-\rho }{\eta \rho }}f_{\gamma_{R_{a}D}}\left(x\right)dx\int_{\frac{x}{\gamma_{th}}}^{\infty }f_{\gamma_{R_{a}E}}\left(y\right)dy\\ & =\int_{0}^{\frac{1-\rho }{\eta \rho }}\exp\left(-\frac{\lambda_{{}_{R_{a}E}}x}{\gamma_{th}}\right)f_{\gamma_{R_{a}D}}\left(x\right)dx\\ \; & =\sum_{k=1}^{N}{\left(-1\right)}^{k+1}C_{N}^{k}k\lambda_{\mathrm{RD}}\int_{0}^{\frac{1-\rho }{\eta \rho }}\exp\left(-k\lambda_{\mathrm{RD}}x-\frac{\lambda_{{}_{R_{a}E}}x}{\gamma_{th}}\right)dx\\ \; & =\sum_{k=1}^{N}{\left(-1\right)}^{k+1}C_{N}^{k}\times \frac{k\lambda_{\mathrm{RD}}}{k\lambda_{\mathrm{RD}}+\frac{\lambda_{{}_{R_{a}E}}}{\gamma_{th}}}\\ \; & \times \left\{1-\exp\left[\frac{\left(\rho -1\right)}{\eta \rho }\left(k\lambda_{\mathrm{RD}}+\frac{\lambda_{{}_{R_{a}E}}}{\gamma_{th}}\right)\right]\right\} \end{array}$$

(33) $$\begin{array}{cc} \Theta_{4} & =\int_{0}^{\frac{1-\rho }{\eta \rho }}f_{\gamma_{R_{a}D}}\left(x\right)dx\int_{0}^{\frac{x}{\gamma_{th}}}f_{\gamma_{R_{a}E}}\left(y\right)dy\int_{0}^{\mu }f_{\gamma_{SR_{a}}}\left(z\right)dz\\ \; & =\int_{0}^{\frac{1-\rho }{\eta \rho }}f_{\gamma_{R_{a}D}}\left(x\right)dx\int_{0}^{\frac{x}{\gamma_{th}}}\lambda_{R_{a}E}\left\{1-\exp(-\lambda_{SR_{a}}\mu )\right\}\times \exp\left(-\lambda_{R_{a}E}y\right)dy\\ \; & =\sum_{k=1}^{N}{\left(-1\right)}^{k+1}C_{N}^{k}k\lambda_{\mathrm{RD}}\lambda_{R_{a}E}\\ & \int_{0}^{\frac{1-\rho }{\eta \rho }}\int_{0}^{\frac{x}{\gamma_{th}}}\left\{1-\exp(-\lambda_{SR_{a}}\mu )\right\}\times \exp\left(-k\lambda_{\mathrm{RD}}x-\lambda_{R_{a}E}y\right)dxdy, \end{array}$$

where $\varphi =\frac{\gamma_{th}-1}{\Phi \left[\left(1-\rho \right)-\gamma_{th}\eta \rho y\right]}$ and $\mu =\frac{\gamma_{th}-1}{\Phi \eta \rho \left[x-\gamma_{th}y\right]}$.

**Theorem** **2.** *By substituting (30)–(33) into (29), SOP can be given as*

(34) $$\begin{array}{c} \mathrm{SOP}=\Theta_{1}+\Theta_{2}+\Theta_{3}+\Theta_{4}. \end{array}$$

**b.**  **Asymptotic Analysis**

In the high signal-to-noise-ratio (SNR) regime, from (34) SOP can be calculated as follows:

(35) $$\begin{array}{cc} {\mathrm{SOP}}^{\Phi \to \infty } & \approx \mathrm{Pr}\left(\frac{\min\left(\left(1-\rho \right)\gamma_{SR_{a}}\Phi ,\eta \rho \Phi \gamma_{SR_{a}}\gamma_{R_{a}D}\right)}{\eta \rho \Phi \gamma_{SR_{a}}\gamma_{R_{a}E}}<\gamma_{th}\right)\\ \; & =\mathrm{Pr}\left(\frac{\min\left(\left(1-\rho \right),\eta \rho \gamma_{R_{a}D}\right)}{\eta \rho \gamma_{R_{a}E}}<\gamma_{th}\right)\\ \; & =\underset{\Theta_{3}}{\underset{︸}{\mathrm{Pr}\left(\gamma_{R_{a}D}<\frac{1-\rho }{\eta \rho },\gamma_{R_{a}E}>\frac{\gamma_{R_{a}D}}{\gamma_{th}}\right)}}\\ & +\underset{\Theta_{1}}{\underset{︸}{\mathrm{Pr}\left(\gamma_{R_{a}D}>\frac{1-\rho }{\eta \rho },\gamma_{R_{a}E}>\frac{1-\rho }{\eta \rho \gamma_{th}}\right)}}. \end{array}$$

**Lemma** **1.** *In the high signal-to-noise-ratio (SNR) regime, the closed-form expression of SOP according to static power splitting-based relaying is expressed as*

(36) $$\begin{array}{cc} {\mathrm{SOP}}^{\Phi \to \infty } & =\sum_{k=1}^{N}{\left(-1\right)}^{k}C_{N}^{k}\exp\left[\frac{\left(\rho -1\right)}{\eta \rho }\left(k\lambda_{\mathrm{RD}}+\frac{\lambda_{{}_{R_{a}E}}}{\gamma_{th}}\right)\right]\\ \; & +\sum_{k=1}^{N}{\left(-1\right)}^{k+1}C_{N}^{k}\times \frac{k\lambda_{\mathrm{RD}}}{k\lambda_{\mathrm{RD}}+\frac{\lambda_{{}_{R_{a}E}}}{\gamma_{th}}}\\ \; & \times \left\{1-\exp\left[\frac{\left(\rho -1\right)}{\eta \rho }\left(k\lambda_{\mathrm{RD}}+\frac{\lambda_{{}_{R_{a}E}}}{\gamma_{th}}\right)\right]\right\}\\ \; & =\sum_{k=1}^{N}{\left(-1\right)}^{k+1}\frac{C_{N}^{k}}{k\lambda_{\mathrm{RD}}+\frac{\lambda_{{}_{R_{a}E}}}{\gamma_{th}}}\\ & \times \left\{k\lambda_{\mathrm{RD}}+\frac{\lambda_{{}_{R_{a}E}}}{\gamma_{th}}\exp\left[\frac{\left(\rho -1\right)}{\eta \rho }\left(k\lambda_{\mathrm{RD}}+\frac{\lambda_{{}_{R_{a}E}}}{\gamma_{th}}\right)\right]\right\}. \end{array}$$

**Proof.**  By substituting (30) and (32) into (35), then (36) is obtained, which finishes the proof. □

### 3.2. Case 2: Dynamic Power Splitting-Based Relaying

In this section, we would like to find the optimal power splitting factor, i.e., $\rho^{*}$ to maximize the system capacity $C_{\mathrm{DF}}$. Because the DF is adopted in our work, $\rho^{*}$ can be calculated as follows:

(37) $$\begin{array}{c} \gamma_{R_{n}}=\gamma_{D}↔\left(1-\rho \right)\gamma_{SR_{n}}\Phi =\eta \rho \Phi \gamma_{SR_{n}}\gamma_{R_{n}D}\to \rho^{*}=\frac{1}{\eta \gamma_{R_{n}D}+1}. \end{array}$$

#### 3.2.1. OP Analysis

**Theorem** **3.** *In dynamic power splitting-based relaying, the closed-form expression of the OP is given as*

(38) $$\begin{array}{cc} OP^{*} & =1-2\sum_{k=1}^{N}{\left(-1\right)}^{k+1}C_{N}^{k}k\exp\left(-\frac{\lambda_{SR_{a}}\gamma_{th}}{\Phi }\right)\\ & \times \sqrt{\frac{k\lambda_{SR_{a}}\lambda_{\mathrm{RD}}\gamma_{th}}{\eta \Phi }}K_{1}\left(2\sqrt{\frac{k\lambda_{SR_{a}}\lambda_{\mathrm{RD}}\gamma_{th}}{\eta \Phi }}\right). \end{array}$$

**Proof.**  By substituting (37) into (22), we have

(39) $$\begin{array}{cc} OP^{*} & =\mathrm{Pr}\left(\frac{\eta \Phi \gamma_{SR_{a}}\gamma_{R_{a}D}}{\eta \gamma_{R_{a}D}+1}<\gamma_{th}\right)=\mathrm{Pr}\left(\gamma_{SR_{a}}<\frac{\gamma_{th}\left(\eta \gamma_{R_{a}D}+1\right)}{\eta \Phi \gamma_{R_{a}D}}\right)\\ \; & =\int_{0}^{\infty }F_{\gamma_{SR_{a}}}\left(\frac{\gamma_{th}\left(\eta x+1\right)}{\eta \Phi x}\right)f_{\gamma_{R_{a}D}}\left(x\right)dx. \end{array}$$

By applying (21), $OP^{*}$ can be rewritten as

(40) $$\begin{array}{cc} OP^{*} & =1-\sum_{k=1}^{N}{\left(-1\right)}^{k+1}C_{N}^{k}k\lambda_{\mathrm{RD}}\int_{0}^{\infty }\exp\left[-\frac{\lambda_{SR_{a}}\gamma_{th}\left(\eta x+1\right)}{\eta \Phi x}\right]\\ & \times \exp\left(-k\lambda_{\mathrm{RD}}x\right)dx\\ \; & =1-\sum_{k=1}^{N}{\left(-1\right)}^{k+1}C_{N}^{k}k\exp\left(-\frac{\lambda_{SR_{a}}\gamma_{th}}{\Phi }\right)\\ & \times \int_{0}^{\infty }\lambda_{\mathrm{RD}}\exp\left(-\frac{\lambda_{SR_{a}}\gamma_{th}}{\eta \Phi x}-k\lambda_{\mathrm{RD}}x\right)dx. \end{array}$$

Finally, by applying ([38], Equation 3.324.1), (38) is obtained. □

#### 3.2.2. SOP Analysis

**a.**  **Exact Analysis**

**Theorem** **4.** *In dynamic power splitting-based relaying, SOP can be expressed as*

(41) $$\begin{array}{cc} {\mathrm{SOP}}^{*} & =1-2\sum_{k=1}^{N}\sum_{t=0}^{\infty }\sum_{m=0}^{t}\frac{{\left(-1\right)}^{k+t+m+1}C_{N}^{k}k\lambda_{\mathrm{RD}}\vartheta^{m}}{m!\left(t-m\right)!{\left(\eta \vartheta \right)}^{t+1}}\\ & \times {\left(\frac{\lambda_{R_{a}E}}{\gamma_{th}}+k\lambda_{\mathrm{RD}}\right)}^{t}G_{1,3}^{3,0}\left(1\left|\begin{array}{c} 0\\ -1,t-m+1,t-m \end{array}\right.\right), \end{array}$$

*where $G_{p,q}^{m,n}\left(\left.z\right|\begin{array}{c} a_{1},...,a_{p}\\ b_{1},...,b_{q} \end{array}\right)$ is the Meijer G-function.*

**Proof.**  From (27) and (37), SOP can be expressed as

(42) $$\begin{array}{cc} {\mathrm{SOP}}^{*} & =\mathrm{Pr}\left(\frac{1+\frac{\eta \Phi \gamma_{SR_{a}}\gamma_{R_{a}D}}{\eta \gamma_{R_{a}D}+1}}{1+\frac{\eta \Phi \gamma_{SR_{a}}\gamma_{R_{a}E}}{\eta \gamma_{R_{a}D}+1}}<\gamma_{th}\right)\\ & =\mathrm{Pr}\left(\frac{\eta \Phi \gamma_{SR_{a}}\gamma_{R_{a}D}}{\eta \gamma_{R_{a}D}+1}<\gamma_{th}-1+\frac{\gamma_{th}\eta \Phi \gamma_{SR_{a}}\gamma_{R_{a}E}}{\eta \gamma_{R_{a}D}+1}\right)\\ \; & =\int_{0}^{\infty }\underset{\Xi }{\underset{︸}{\mathrm{Pr}\left(\eta \Phi \gamma_{SR_{a}}x<\left(\gamma_{th}-1\right)\left(\eta x+1\right)+\gamma_{th}\eta \Phi \gamma_{SR_{a}}\gamma_{R_{a}E}\right)}}\\ & \times f_{\gamma_{R_{a}D}}\left(x\right)dx\\ & =\sum_{k=1}^{N}{\left(-1\right)}^{k+1}C_{N}^{k}k\lambda_{\mathrm{RD}}\int_{0}^{\infty }\Xi \times \exp\left(-k\lambda_{\mathrm{RD}}x\right)dx, \end{array}$$

where Ξ can be calculated as

(43) $$\begin{array}{cc} \Xi & =\mathrm{Pr}\left(\eta \Phi \gamma_{SR_{a}}x<\left(\gamma_{th}-1\right)\left(\eta x+1\right)+\gamma_{th}\eta \Phi \gamma_{SR_{a}}\gamma_{R_{a}E}\right)\\ \; & =\mathrm{Pr}\left(\gamma_{SR_{a}}\left[\eta \Phi x-\gamma_{th}\eta \Phi \gamma_{R_{a}E}\right]<\left(\gamma_{th}-1\right)\left(\eta x+1\right)\right)\\ \; & =\left\{\begin{array}{c} \mathrm{Pr}\left(\gamma_{SR_{a}}<\frac{\left(\gamma_{th}-1\right)\left(\eta x+1\right)}{\eta \Phi x-\gamma_{th}\eta \Phi \gamma_{R_{a}E}}\right),\gamma_{R_{a}E}>\frac{x}{\gamma_{th}}\\ 1,\;\;\;\;\gamma_{R_{a}E}\leq \frac{x}{\gamma_{th}} \end{array}\right. \end{array}$$

(44) $$\begin{array}{cc} \; & =\int_{0}^{\frac{x}{\gamma_{th}}}f_{\gamma_{R_{a}E}}\left(y\right)dy+\int_{\frac{x}{\gamma_{th}}}^{\infty }F_{\gamma_{SR_{a}}}\left[\frac{\left(\gamma_{th}-1\right)\left(\eta x+1\right)}{\eta \Phi x-\gamma_{th}\eta \Phi y}\right]\times f_{\gamma_{R_{a}E}}\left(y\right)dy\\ \; & =1-\lambda_{R_{a}E}\int_{\frac{x}{\gamma_{th}}}^{\infty }\exp\left[-\frac{\lambda_{SR_{a}}\left(1-\gamma_{th}\right)\left(\eta x+1\right)}{\gamma_{th}\eta \Phi y-\eta \Phi x}-\lambda_{R_{a}E}y\right]dy. \end{array}$$

By denoting $u=\gamma_{th}\eta \Phi y-\eta \Phi x$, (43) can be rewritten by

(45) $$\begin{array}{cc} \Xi & =1-\frac{\lambda_{R_{a}E}}{\gamma_{th}\eta \Phi }\\ & \int_{0}^{\infty }\exp\left[-\frac{\lambda_{SR_{a}}\left(1-\gamma_{th}\right)\left(\eta x+1\right)}{u}-\frac{\lambda_{R_{a}E}\left(\eta \Phi x+u\right)}{\gamma_{th}\eta \Phi }\right]du\\ \; & =1-\frac{\lambda_{R_{a}E}}{\gamma_{th}\eta \Phi }\exp\left(-\frac{\lambda_{R_{a}E}x}{\gamma_{th}}\right)\\ & \int_{0}^{\infty }\exp\left[-\frac{\lambda_{R_{a}E}u}{\gamma_{th}\eta \Phi }-\frac{\lambda_{SR_{a}}\left(1-\gamma_{th}\right)\left(\eta x+1\right)}{u}\right]du. \end{array}$$

By applying ([38] Equation 3.324.1), Ξ can be obtained as

(46) $$\begin{array}{cc} \Xi & =1-2\exp\left(-\frac{\lambda_{R_{a}E}x}{\gamma_{th}}\right)\sqrt{\frac{\lambda_{SR_{a}}\lambda_{R_{a}E}\left(1-\gamma_{th}\right)\left(\eta x+1\right)}{\gamma_{th}\eta \Phi }}\\ & \times K_{1}\left(2\sqrt{\frac{\lambda_{SR_{a}}\lambda_{R_{a}E}\left(1-\gamma_{th}\right)\left(\eta x+1\right)}{\gamma_{th}\eta \Phi }}\right). \end{array}$$

By substituting (47) into (42), we have

(47) $$\begin{array}{cc} {\mathrm{SOP}}^{*} & =\sum_{k=1}^{N}{\left(-1\right)}^{k+1}C_{N}^{k}k\lambda_{\mathrm{RD}}\int_{0}^{\infty }(1-2\exp\left(-\frac{\lambda_{R_{a}E}x}{\gamma_{th}}\right)\\ & \sqrt{\vartheta (\eta x+1)}\times K_{1}\left(2\sqrt{\vartheta (\eta x+1)}\right))\times \exp\left(-k\lambda_{\mathrm{RD}}x\right)dx\\ \; & =1-2\sum_{k=1}^{N}{\left(-1\right)}^{k+1}C_{N}^{k}k\lambda_{\mathrm{RD}}\int_{0}^{\infty }\exp\left(-\left(\frac{\lambda_{R_{a}E}}{\gamma_{th}}+k\lambda_{\mathrm{RD}}\right)x\right)\\ & \times \sqrt{\vartheta (\eta x+1)}\times K_{1}\left(2\sqrt{\vartheta (\eta x+1)}\right)dx, \end{array}$$

where $\vartheta =\frac{\lambda_{SR_{a}}\lambda_{R_{a}E}\left(1-\gamma_{th}\right)}{\gamma_{th}\eta \Phi }$.

By applying the Taylor series $\exp\left(-\left(\frac{\lambda_{R_{a}E}}{\gamma_{th}}+k\lambda_{\mathrm{RD}}\right)x\right)=\sum_{t=0}^{\infty }\frac{{\left(-1\right)}^{t}}{t!}{\left(\frac{\lambda_{R_{a}E}}{\gamma_{th}}+k\lambda_{\mathrm{RD}}\right)}^{t}x^{t}$ and denoting y=ϑ(ηx+1), SOP can be calculated as

(48) $$\begin{array}{cc} {\mathrm{SOP}}^{*} & =1-2\sum_{k=1}^{N}\sum_{t=0}^{\infty }\frac{{\left(-1\right)}^{k+1+t}C_{N}^{k}k\lambda_{\mathrm{RD}}}{t!{\left(\eta \vartheta \right)}^{t+1}}\\ & {\left(\frac{\lambda_{R_{a}E}}{\gamma_{th}}+k\lambda_{\mathrm{RD}}\right)}^{t}\int_{1}^{\infty }{\left(y-\vartheta \right)}^{t}\times \sqrt{y}\times K_{1}\left(2\sqrt{y}\right)dy. \end{array}$$

By applying ${\left(x+y\right)}^{t}=\sum_{m=0}^{t}\frac{t!}{m!(t-m)!}x^{t-m}y^{m}$, we have

(49) $$\begin{array}{cc} {\mathrm{SOP}}^{*} & =1-2\sum_{k=1}^{N}\sum_{t=0}^{\infty }\sum_{m=0}^{t}\frac{{\left(-1\right)}^{k+t+m+1}C_{N}^{k}k\lambda_{\mathrm{RD}}\vartheta^{m}}{m!\left(t-m\right)!{\left(\eta \vartheta \right)}^{t+1}}\\ & {\left(\frac{\lambda_{R_{a}E}}{\gamma_{th}}+k\lambda_{\mathrm{RD}}\right)}^{t}\int_{1}^{\infty }y^{t-m+1/2}\times K_{1}\left(2\sqrt{y}\right)dy. \end{array}$$

Finally, by using ([38], Equation 6.592.4), SOP can be obtained as in (41). □

**b.**  **Asymptotic Analysis**

From (41), SOP can be approximated as

(50) $$\begin{array}{cc} {\mathrm{SOP}}_{\Phi \to \infty }^{*} & \approx \mathrm{Pr}\left(\frac{\gamma_{R_{a}D}}{\gamma_{R_{a}E}}<\gamma_{th}\right)=\mathrm{Pr}\left(\gamma_{R_{a}D}<\gamma_{th}\gamma_{R_{a}E}\right)\\ \; & =\int_{0}^{\infty }F_{\gamma_{R_{a}D}}\left(\gamma_{th}x\right)\times f_{\gamma_{R_{a}E}}\left(x\right)dx\\ & =1+\sum_{k=1}^{N}{\left(-1\right)}^{k}C_{N}^{k}\int_{0}^{\infty }\exp\left(-k\lambda_{\mathrm{RD}}x-\lambda_{R_{a}E}x\right)dx\\ \; & =1+\sum_{k=1}^{N}\left[\frac{{\left(-1\right)}^{k}C_{N}^{k}}{k\lambda_{\mathrm{RD}}+\lambda_{R_{a}E}}\right]. \end{array}$$

## 4. Simulation Results

In this section, we present the proposed partial relay selection in terms of the outage probability and secrecy outage probability via analysis and simulation results. All transmission links are Rayleigh fading channels, and the path-loss model is considered, where the path loss exponent equals 2.5. The locations of source S, relay R, destination D, and eavesdropper E are (0,0), (0.5, 0), (2, 0), and (0.5, 2), respectively. To obtain the outage probability and secrecy outage probability for the proposed methods, we execute $10^{6}$ independent samples, and the channel coefficients are randomly generated as Rayleigh fading in each sample.

**Figure 2:**
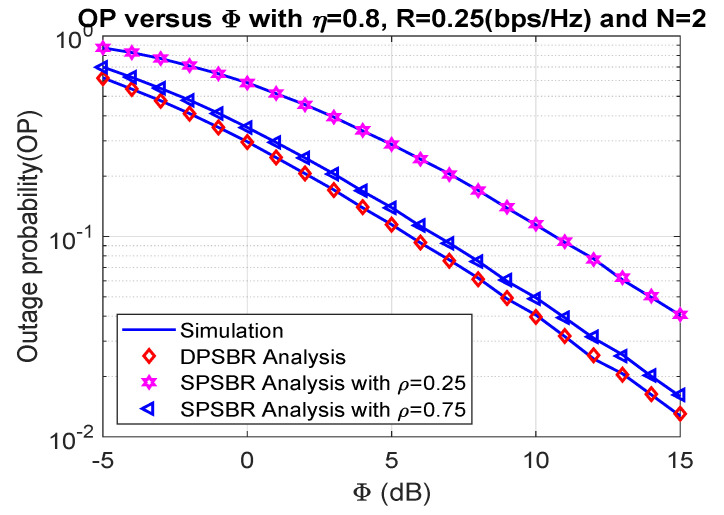
Outage probability versus Φ (dB).

**Figure 3:**
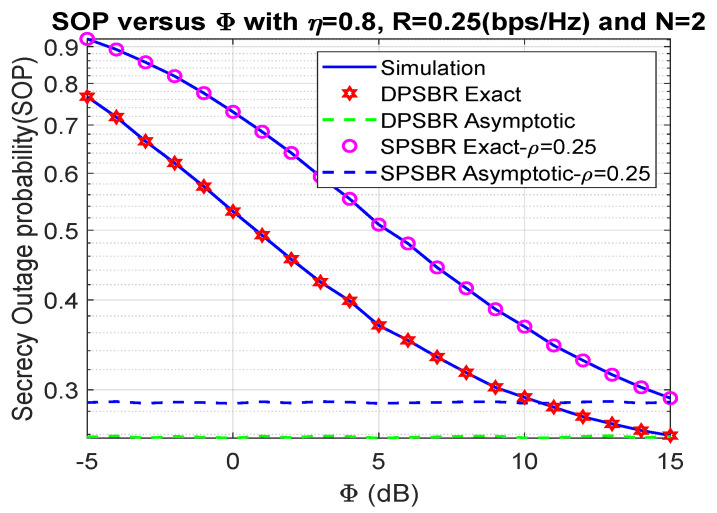
Outage probability versus Φ (dB).

In Figure 2 and Figure 3, we show the impact of Φ on the OP and SOP, where η= 0.8, R= 0.25 bps/Hz, and N = 2. In Figure 2 and Figure 3, we compared the dynamic power splitting-based relaying (DPSBR) with the static power splitting-based relaying (SPSBR), whereas the SPSBR is considered in two-mode ρ equals 0.25 and 0.75, respectively. First, it is easy to see that the DPSBR obtains better OP and SOP results compared to SPSBR methods. Specifically, when Φ = 15 dB, the OP of DPSBR is 0.013, while the SPSBR with ρ=0.25 and ρ=0.75 impose 0.0407 and 0.0159, respectively. This is because the DPSBR scheme aims to maximize the system capacity, thus it can improve the outage performance while the SPSBR scheme always select a fixed value of power splitting factor ρ. Second, the higher the Φ value is, the better OP and SOP can be obtained. It can be explained by the fact that the higher Φ value means the more transmit power of source S is assigned, which is defined in Equation (11).

**Figure 4:**
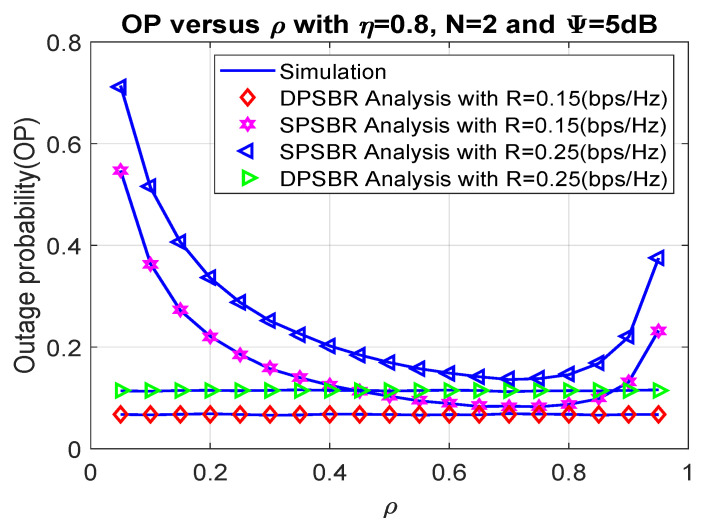
Outage probability versus ρ.

**Figure 5:**
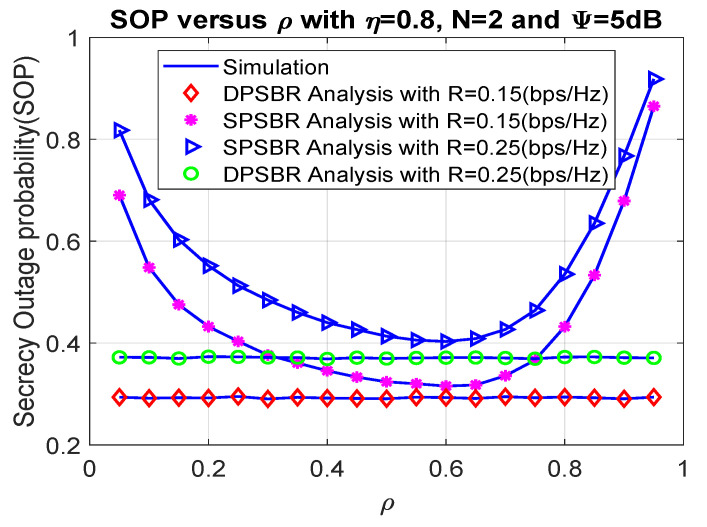
Secrecy outage probability versus ρ.

Next, we investigate the OP and SOP subject to different power splitting factor ρ in Figure 4 and Figure 5, where η= 0.8, number of relays N = 2, and Ψ = 5 dB. The power splitting factor ρ plays an important role as it affects the fraction of power used for energy harvesting and data transmission. Therefore, there exists an optimal value of power splitting factor to maximize the outage probability and secrecy outage probability. Specifically, the SPSBR with R = 0.15 bps/Hz and R = 0.25 bps/Hz can obtain the best outage value at ρ = 0.7, and their OP/SOP values result in a parabolic shape. Notably, it is shown from Figure 4 and Figure 5 that the OP and SOP of the DPSBR do not depend on ρ value. This is because the DPSBR is designed to be used at the best ρ value and it is fixed when we operate the system.

**Figure 6:**
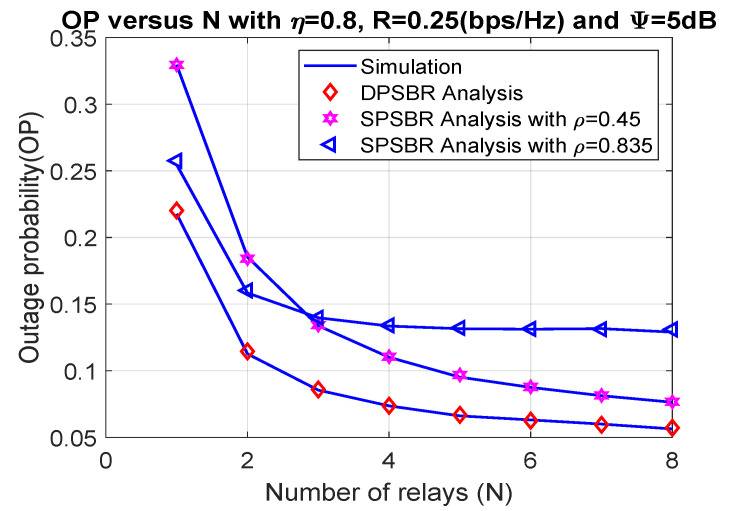
Outage probability versus number of relays (N).

**Figure 7:**
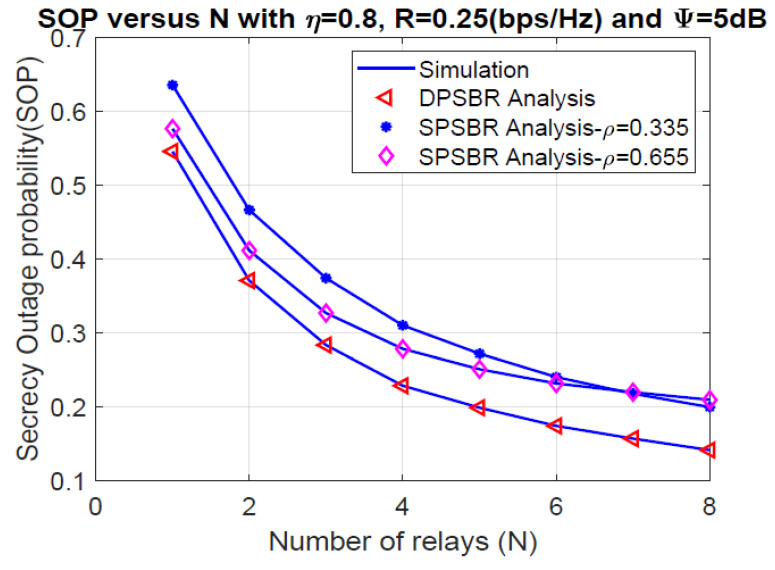
Secrecy outage probability versus number of relays (N).

Last, Figure 6 and Figure 7 plot the OP and SOP as functions of number of relays (N), where η = 0.8, R = 0.25 bps/Hz, and Ψ= 5 dB. It is observed that the proposed DPSBR method outperforms other benchmark ones, i.e., SPSBR with ρ = 0.45 and SPSBR with ρ = 0.835. More specifically, when N = 8, the OP of DPSBR scheme is 0.0571, while the SPSBR with ρ = 0.45 and SPSBR with ρ = 0.835 impose 0.0767 and 0.129, respectively. Particularly, the SPSBR with ρ = 0.835 can obtain a better outage performance compared to SPSBR with ρ = 0.45 with a low number of relays, i.e., N < 3. However, when the number of relays is large enough, i.e., N > 3, SPSBR with ρ = 0.835 deteriorates than SPSBR with ρ = 0.45. In Figure 7, we study the effect of number of relays on SOP. It is revealed that increasing the number of relays significantly improves the secrecy performance. This is because the higher the number of relays is, the better the channel selection from relay to destination is, which enhances the secrecy performance.

## 5. Conclusions

This paper proposed a partial relay selection scheme for SWIPT-based HD DF relaying under the presence of an eavesdropper. Specifically, we investigated the OP and SOP for dynamic power splitting-based relaying and statistic power splitting-based relaying. Most importantly, the closed-form expressions of OP and SOP (i.e., for exact and asymptotic analysis) are derived. Mote Carlo simulations were given to demonstrate the correctness of our theoretical analysis. In general, the proposed DPSBR scheme showed its superiority compared to SPSBR in terms of OP and SOP. More specifically, extensive simulation results showed that the OP and SOP performance of the DPSBR can improve up 94.5% and 33.4% than SPSBR schemes, respectively. In particular, when the power splitting factor ρ equals 0.5, the OP and SOP values of the SPSBR scheme with R = 0.15 bps/Hz obtained a performance almost DPSBR. Therefore, the system should operate in the SPSBR scheme in this scenario for a simple implementation.

In future work, it will be interesting to extend this work to the following research directions: (1) The relay users can be UAVs or intelligent reflecting surfaces; (2) Using a friendly jammer or artificial noise to improve the system security; (3) A more general system model such as an independent but not identically distributed Rayleigh fading or Nakagami-m fading channel.
